# Same-Day HIV Pre-Exposure Prophylaxis (PrEP) Initiation During Drop-in Sexually Transmitted Diseases Clinic Appointments Is a Highly Acceptable, Feasible, and Safe Model that Engages Individuals at Risk for HIV into PrEP Care

**DOI:** 10.1093/ofid/ofz310

**Published:** 2019-06-27

**Authors:** Kevin F Kamis, Grace E Marx, Kenneth A Scott, Edward M Gardner, Karen A Wendel, Mia L Scott, Angela E Montgomery, Sarah E Rowan

**Affiliations:** 1 Denver Public Health, Denver Health and Hospital Authority, Colorado; 2 University of Colorado, Department of Medicine, Division of Infectious Diseases, Aurora; 3 Colorado School of Public Health, Department of Epidemiology, Aurora; 4 Apex Family Medicine, Denver, Colorado

**Keywords:** same-day PrEP, STD clinics

## Abstract

**Objective:**

Strategies to increase pre-exposure prophylaxis (PrEP) uptake are needed. We hypothesized that same-day PrEP initiation in a sexually transmitted diseases (STD) clinic would be acceptable, feasible, and safe, and that individuals would engage in ongoing PrEP care.

**Method:**

Individuals aged ≥ 18 years were evaluated for PrEP. Exclusion criteria were HIV, history of renal dysfunction or chronic hepatitis B infection, pregnancy, indications for HIV post-exposure prophylaxis, or positive screen for acute HIV symptoms. One hundred individuals received a free 30-day PrEP starter pack and met with a patient navigator to establish ongoing care. Bivariate analysis and multivariable logistic regression were used to compare individuals who did and did not attend at least 1 PrEP follow-up appointment within 180 days of enrollment. Client satisfaction surveys were given 3 months after enrollment.

**Results:**

The majority (78%) of participants completed at least 1 PrEP follow-up appointment, and 57% attended at least 2 follow-up appointments. After adjusting for race and ethnicity, age, health insurance status, and annual income, only income was associated with follow-up appointment attendance. Each additional $10,000 increase in income was associated with a 1.7-fold increase in the odds of attending a PrEP follow-up appointment (95% confidence interval, 1.07–2.66, *P* = .02). The majority (54%) of individuals completed the satisfaction survey and all respondents liked the option of same-day PrEP initiation.

**Conclusions:**

Our study suggests STD clinic-based, same-day PrEP initiation is acceptable, feasible, safe, and links a high proportion of individuals into ongoing PrEP care. Additional resources may be needed to support low-income individuals’ retention in care.

## Teaser

The majority (78%) of individuals initiated on same-day pre-exposure prophylaxis (PrEP) in an sexually transmitted diseases clinic completed at least 1 follow-up appointment. Income was positively associated with follow-up appointment attendance. Our study suggests same-day PrEP initiation is safe, feasible, and well-received.

## INTRODUCTION

With almost 40 000 new diagnoses in the US in 2017, HIV remains a critical public health priority [1]. Pre-exposure prophylaxis (PrEP) with daily oral coformulated tenofovir disoproxil fumarate and emtricitabine (TDF/FTC) has demonstrated effectiveness in the prevention of HIV acquisition among men who have sex with men (MSM) and transgender women [2], heterosexual individuals [3, 4], and people who inject drugs [5]. More than 1.2 million persons in the US are estimated to have indications for PrEP [6], yet fewer than 50 000 individuals initiated PrEP between 2013 and 2015 [7], underscoring the vital need for new strategies to increase PrEP uptake.

Sexually transmitted disease (STD) clinics are a healthcare venue with great potential for increasing PrEP initiations among individuals at increased risk of HIV acquisition and reaching populations with limited contact with the health care system [8–11]. Individuals attending STD clinics have high reported interest in PrEP [12, 13], and a PrEP demonstration project integrated with STD care showed good PrEP uptake and adherence [14]. A recent modeling study suggested a greater reduction of HIV incidence through PrEP provision to MSM through a STD clinic-based PrEP delivery model compared to PrEP delivery to MSM recruited from the community [15].

STD clinics may face challenges to PrEP care provision given that they frequently lack capacity for continuity care, toxicity monitoring, and adherence support [8, 10, 11]. For these reasons, STD clinics often rely on referral-based models for PrEP care in which the STD clinic refers individuals eligible for PrEP to outside clinics for PrEP initiation, laboratory monitoring, and clinical follow-up. Given the inevitable time lag and logistic challenges of scheduling an appointment at an outside clinic, referral-based models increase the time an individual remains at risk for HIV acquisition and often result in individuals who are lost to follow-up between PrEP referral and PrEP initiation [16, 17].

We sought to determine whether a same-day approach to PrEP initiation could be feasible, acceptable, and successfully implemented in a busy, urban, and public STD clinic. We hypothesized that same-day PrEP initiation would be safe and convenient, well received by clients, and that a high proportion of individuals initiated on same-day PrEP would engage in ongoing PrEP care.

## METHODS

### Setting

This study was conducted at the Denver Metro Health Clinic (DMHC), the largest STD clinic in metropolitan Denver, Colorado. DMHC is part of the Denver Health system, an integrated safety-net health care system, and it provides sexual health services at low or no cost, primarily through walk-in visits that do not require an advance appointment.

### Study Population

Inclusion criteria were individuals aged ≥ 18 years presenting for care at DMHC and meeting indications for PrEP as defined by the Centers for Disease Control and Prevention [18]. Exclusion criteria were history of renal dysfunction, HIV, or a history of chronic hepatitis B virus infection, pregnancy, indications for HIV post-exposure prophylaxis, or signs and symptoms consistent with acute HIV. Individuals were ineligible to participate if they could not follow-up for ongoing PrEP care at 1 of the participating clinics. To participate in the study, individuals had to be willing to have additional blood tests and meet with the patient navigator the same day of their clinical visit. Consecutive eligible individuals were enrolled until the enrollment limit of 100 study enrollees was reached. This number was determined by availability of funding for PrEP starter packs.

### Study Protocol

Individuals were evaluated for PrEP eligibility by a nurse practitioner (NP) or by a registered nurse with attending physician oversight, and readiness to initiate same-day PrEP and pursue ongoing care at a participating clinic was established by a consensus between these providers and a patient navigator.

Interested individuals underwent laboratory screening, including serum creatinine, hepatitis B surface antigen, urine pregnancy test, and point-of-care HIV antigen/antibody test (Determine HIV-1/2 Antigen/Antibody Combo; Abbott, Abbott Park, IL). Individuals were assessed for a history of hepatitis B virus infection or renal disease. HIV antigen/antibody and pregnancy test results were reviewed during the visit. All other laboratory test results were reviewed by an NP; serum creatinine results were available in 1 day and hepatitis B surface antigen results were available in 2 days on average. HIV viral load testing was available at the attending physician’s discretion. A protocol was established to contact individuals with abnormal lab results within 2 days of enrollment.

Eligible and interested individuals were offered same-day PrEP initiation. An on-site pharmacist provided each enrollee with a free 30-day supply of TDF/FTC as well as adherence and potential medication adverse events counseling. Individuals were instructed to call DMHC if concerned about medication side effects or to present to an emergency room or urgent care center if experiencing severe adverse reactions. Individuals interested in PrEP but not same-day initiation were offered a standard PrEP referral to outside clinics.

Study enrollees met with a patient navigator who provided PrEP education and confirmed readiness to start same-day PrEP. The navigator conducted a financial screen assessing for current household income and insurance status. Uninsured or under-insured individuals eligible for study enrollment were referred to an insurance enrollment specialist, a manufacturer-sponsored financial assistance program, and a state-sponsored financial assistance program as needed.

The patient navigator scheduled 1-month follow-up appointments at a participating clinic according to client preference and insurance. Participating clinics included 9 primary care clinics (all federally qualified health centers) within the Denver Health system, 2 local infectious diseases clinics (1 within the Denver Health system and 1 within the local university health system), and a local private practice internal medicine clinic.

The patient navigator called all study participants 1 week after enrollment to assess for medication side effects. Clinical questions were addressed by a DMHC staff physician. Patient navigation of the healthcare and insurance systems was provided as needed until the first PrEP follow-up appointment. Individuals were given the contact information of the patient navigator.

Upon enrollment, sex, gender, race, ethnicity, age, annual income, health insurance status, primary care provider status, risk factors for HIV acquisition, and history of confirmed bacterial STD diagnoses (chlamydia, gonorrhea, and syphilis) within 180 days prior to or at the time of enrollment were recorded. Participants’ medical records were reviewed for documentation of attendance to follow-up PrEP appointments at participating clinics up to 180 days after enrollment.

A follow-up client satisfaction survey with 9 quantitative questions and 5 open-ended questions was given to all individuals 3 months after enrollment to assess satisfaction with same-day PrEP services (see Supplementary Data for follow-up survey questionnaire and quantitative responses). Individuals were able to self-administer the survey electronically or complete the survey over the phone with the study coordinator.

The study was approved by the Colorado Multiple Institutional Review Board.

### Data Analysis

To assess utilization of same-day PrEP initiation services among clients who discussed PrEP with an STD clinic provider, we quantified all STD visits during the 6 month study period in which PrEP was discussed. The study population was characterized using descriptive statistics. Bivariate analysis and multivariable logistic regression were used to compare individuals who did and did not attend at least 1 PrEP follow-up visit appointment. All statistical analyses were performed in SAS/STAT software, version 7.1 (SAS Institute Inc., Cary, NC).

## RESULTS

### Study Population

**Figure 1.**
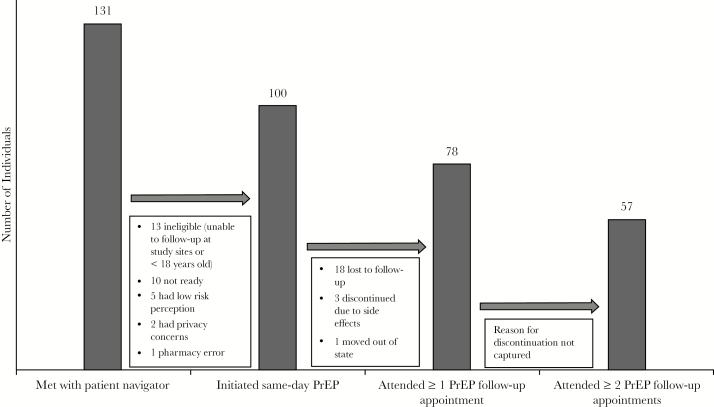
Follow-Up PrEP Care Cascade After Same-Day Initiation

Between April 1, 2017, and October 4, 2018, 131 participants were referred by DMHC clinicians to the patient navigator to be screened for study enrollment. This represents 22% of the 584 individuals who discussed PrEP with a provider in the STD clinic during this time. One hundred individuals were enrolled into the study (Figure 1).

Median participant age was 28 years (interquartile range [IQR], 25–33 years), 98% were cis-gender male, and 2% were cis-gender female. About half of all participants (48%) were non-Hispanic white, 39% were Hispanic, and 8% were non-Hispanic black. At enrollment, reported median annual income was $24 000 (IQR, $14 000–$38 000), 62% had health insurance (including Medicaid), 26% had a primary care provider, and 50% had a history of recent bacterial STD (chlamydia, gonorrhea, or syphilis) (Table 1).

**Table 1.** Comparison of Individuals Who Did and Did Not Attend At Least One PrEP Follow-Up Appointment After Receiving PrEP Starter Pack

	Attended ≥ 1 Follow-up Appointment	Did Not Attend Follow-up Appointment	
Characteristics	(n = 78)	(n = 22)	*P* Value^e^
Gender			
Male	76 (97%)	22 (100%)	1.00
Female	2 (3%)	0 (0%)	
Race/ethnicity			
White	40 (51%)	8 (36%)	.22
Black	5 (6%)	3 (14%)	
Hispanic (all races)	30 (38%)	9 (41%)	
Asian	2 (3%)	2 (9%)	
Other	1 (1%)	0 (0%)	
Age (years)			
18–29	42 (54%)	17 (77%)	.05
≥30	36 (46%)	5 (23%)	
Annual income^a^			
≤ 133% FPL^b^	18 (24%)	12 (57%)	.01
134%–300% FPL	35 (47%)	8 (38%)	
>300% FPL	21 (28%)	1 (5%)	
Health insurance status at enrollment^c^			
Insured	54 (69%)	8 (36%)	.01
Uninsured	24 (31%)	14 (64%)	
Primary care provider at enrollment			
Yes	25 (32%)	1 (5%)	.01
No	53 (68%)	21 (95%)	
History of recent bacterial STD^d^			
Yes	38 (49%)	12 (55%)	.63
No	40 (51%)	10 (45%)	

Date represent number (%) of participants unless otherwise indicated.

Abbreviations: FPL, Federal Poverty Level; IQR, interquartile range; STD, sexually transmitted disease.

^a^Five individuals preferred not to report income.

^b^133% of the Federal Poverty Level (FPL) was the cut off for eligibility for Colorado’s Medicaid program at the time of our study. In 2017, 133% FPL for a single individual without dependent children equated to an annual income of $16 044, and 300% FPL for a singled individual without dependent children equated to annual income of $36 180.

^c^Defined as having any type of health insurance.

^d^Defined as having gonorrhea, chlamydia, or syphilis verified by electronic health records within the previous 180 days or at enrollment.

^e^Fischer exact test was used for gender comparison; all other analyses were chi-square test. Race was collapsed to white vs non-white.

Regarding indications for PrEP, all 98 male participants were MSM, 69% of whom reported condomless anal intercourse within the 6 months prior to enrollment. Among all participants, 19% reported having a recent sexual partner living with HIV, 14% reported ever having been on PrEP prior to the study, 5% reported previous use of HIV post-exposure prophylaxis for sexual exposures, 1% reported current injection drug use, and 1% reported ever engaging in commercial sex work.

### Baseline Laboratory Results

No participant had abnormal baseline laboratory results. Creatinine clearance was normal in all individuals tested. One individual did not have creatinine tested upon PrEP initiation due to clinic error, but was later tested and the result was within normal limits. No individual tested positive for hepatitis B virus surface antigen at baseline. One HIV viral load test was ordered and the result was negative (no HIV RNA detected).

### Patient Navigation

All 100 participants received financial assessment and the majority (65%) linked to at least 1 PrEP financial assistance program. Almost a quarter (23%) of all individuals were referred to an insurance enrollment specialist. The majority of participants (55%) were referred to a primary care clinic, and 45% were referred to an infectious disease clinic.

### Follow-Up Appointment Attendance

The majority (78%) of participants attended at least 1 follow-up visit with a PrEP provider and 76% of those who completed their first follow-up visit did so within 31 days of enrollment (median time to fist follow-up appointment, 28 days; IQR, 23–31 days) (Figure 1). More than half (57%) of all individuals attended at least 2 follow-up appointments with a PrEP provider within 180 days of enrollment (median time to second-follow-up appointment, 109 days; IQR, 91–129 days). No HIV seroconversions were detected by medical chart review during the 6 month follow-up period.

In bivariate analysis, older age, higher annual income, reported health insurance status coverage at enrollment, and having a primary care provider had a positive statistically significant association (*P* < .05) with attending at least 1 PrEP follow-up appointment (Table 1). In a multivariable logistic regression model, after adjusting for race/ethnicity, age, health insurance status at enrollment, and income, only income was associated with attending a PrEP appointment (Table 2). Each additional $10 000 increase in annual income was associated with a 1.7 fold increase in the odds of attending at least 1 PrEP follow-up appointment (95% CI, 1.07–2.66; *P* = .02). No other covariate was statistically significant in the final regression model.

**Table 2.** Multivariable Regression Model Comparing Characteristics of Individuals Who Did and Did Not Attend At Least One PrEP Follow-Up Appointment

Characteristic	Crude OR (95% CI)	*P* Value	Adjusted OR (95% CI)	*P* Value
**Age** (units = 10 years)	2.07 (0.90–4.73)	.09	2.09 (0.78–5.59)	.14
**Race/ethnicity**				
White	REF	—	REF	—
Non-white	0.54 (0.21–1.44)	.22	0.87 (0.29–2.63)	.81
**Annual income** (units = $10 000)	1.84 (1.22–2.77)	<.01	1.69 (1.07–2.66)	.02
**Health insurance status at enrollment**				
Insured	REF	—	REF	—
Uninsured	0.25 (0.09–0.69)	.01	0.39 (0.13–1.19)	.10

Abbreviations: CI, confidence interval; OR, odds ratio.

### Client Satisfaction Follow-Up Survey

Fifty-four of the 100 study participants responded to the follow-up client satisfaction survey. All respondents (100%) reported that they liked having the option of same-day PrEP initiation. The majority of respondents (96%) reported that they plan on continuing to take PrEP. A minority of patients (13%) reported difficulty with PrEP initiation, including mild to moderate side effects and insurance challenges (see Supplementary Data for full quantitative survey response frequencies). In the qualitative survey questions, individuals stated that the service was convenient, easy, and removed important barriers to starting PrEP, such as requiring multiple visits. Suggestions for improvement included reduced paperwork required for financial assistance program applications and shortened overall visit time.

## DISCUSSION

Almost 80% of the 100 individuals starting same-day HIV PrEP in a walk-in STD clinic attended at least 1 follow-up PrEP appointment, and almost 60% attended at least 2 follow-up PrEP appointments within 180 days of initiation. The model was well received by study participants who completed the client satisfaction follow-up survey. Our study demonstrated that STD clinic-based, same-day PrEP initiation with simultaneous patient navigation can serve as a safe and convenient entryway into ongoing PrEP care and is a model that was well received by STD clinic clients.

Our findings are consistent with a recent study of immediate PrEP initiation at sexual health clinics in New York City that demonstrated safety of immediate PrEP initiation at walk-in sexual health clinics [19]. A growing body of literature has also supported rapid initiation of antiretroviral therapy for individuals with newly diagnosed HIV. These studies have demonstrated high patient acceptance, high rates of linkage to care, earlier viral suppression, and increased retention in care [20–23]. Rapid start models are thus promising approaches to address gaps and challenges of traditional HIV treatment and prevention models.

The same-day PrEP initiation model presented here addresses both delayed time to PrEP initiation and low linkage to care rates found in traditional PrEP models. A recent study of a primary care safety-net healthcare system in San Francisco found that almost 30% of individuals had a wait time of more than 30 days before initiating PrEP [24]. A referral-based model in public STD clinics in Chicago found that only 29% of individuals eligible for active referral received a PrEP prescription [17], and 34% of individuals accepting active PrEP referral through a PrEP navigation program at sexual health clinics in New York City were prescribed PrEP from an external PrEP provider [25]. In 2015 at DMHC, only 47% of the 177 individuals actively referred for PrEP care completed a PrEP intake visit. STD clinic-based, same-day PrEP initiation models therefore not only reduce time until a PrEP-eligible and interested client can start PrEP, but they may also lead to a higher proportion of individuals linked to ongoing PrEP care compared to referral-only models.

In the medical community, 1 area of concern for same-day PrEP programs has been the potential that important abnormal baseline laboratory findings (hepatitis B status and renal function) could lead to increased risk for poor outcomes. In our study, no individual was found to have a reactive hepatitis B surface antigen or an elevated creatinine level at baseline, indicating the low prevalence of previously unrecognized renal disease and active hepatitis B virus infection among individuals accessing same-day PrEP services in our local STD clinic environment. Although the sample size was relatively small, this supports the general safety of same-day HIV PrEP initiation in the absence of point-of-care hepatitis serologies or serum creatinine testing. Furthermore, there are studies that have shown the safety of HIV PrEP initiation even in the setting of hepatitis B virus infection [26] and the general reversibility of glomerular renal function decline upon discontinuation of TDF [27]. Screening for hepatitis B virus infection and abnormal renal function will need to be carried out and followed closely in PrEP programs. However, at least in our environment, it appears that critical abnormalities will be relatively uncommon.

Same-day PrEP initiation requires immediate access to HIV test results. The CDC guidelines for HIV PrEP recommend documentation of a negative test result within a week before initiation of PrEP, ideally with a laboratory-based antigen/antibody test [18]. Many STD clinics, including ours, use a point-of-care antigen/antibody test. The window period of point-of-care tests is longer than laboratory-based tests in some individuals [28]. To address this, we screened for signs and symptoms of acute HIV prior to initiation and deferred PrEP initiation if acute HIV was suspected. HIV viral load testing was available at the attending physician’s discretion. In addition, the initial TDF/FTC prescription was for 30 days, which required individuals to have a follow-up PrEP appointment with a provider for repeat HIV testing within 1 month of initiation prior to medication refill, as recommended by the International Antiviral Society–USA Panel [29], to diagnose anyone who might have been in the window period at the time of PrEP initiation.

The majority of individuals required financial assistance to cover PrEP costs in our study. At the time of our study, a manufacturer prescription co-pay assistance program covered up to $3600 of out-of-pocket prescription copay costs for commercially insured individuals and covered all prescription costs for uninsured individuals. During this same time period, a state-sponsored PrEP financial assistance program in Colorado covered PrEP-related medical visit and lab costs for both uninsured and insured individuals who earned up to 500% of the Federal Poverty Line ($60 300 per year for a single individual in 2017).

Despite the provision of financial navigation for every study enrollee and the availability of financial assistance programs to cover out of pocket costs associated with PrEP prescription and medical care costs, low income was the major finding associated with not linking to ongoing PrEP care. We speculate competing needs faced by individuals with low incomes impact engagement in ongoing care. Both real and perceived costs directly related to PrEP care have been shown to negatively impact retention [30, 31], but more research is needed to elucidate which barriers continue to impact low income individuals’ engagement in ongoing PrEP care after out-of-pocket costs are covered.

This study has limitations. First, the number of eligible individuals and refusals to meet with the study coordinator were not captured and, thus, we are unable to calculate acceptance rate. Our study sample was similar in age distribution to our general STD clinic population, but we had a smaller proportion of black study participants in the study sample (8%) than were seen in the STD clinic during the study period (17%). The lack of a comparison group limits generalizability of this approach to all PrEP-eligible candidates at the clinic during the study period, and the sample of individuals who elected to initiate same-day PrEP is potentially biased. Furthermore, the high follow-up rate seen in our study participants may not be attained in other settings. More research is needed to assess the potential reach of same-day PrEP among PrEP-eligible clients across different settings and subgroups.

Certain financial assistance programs utilized by many individuals in this study are specific to Colorado and might not be generalizable to other settings. The provision of free starter packs allowed all participants to immediately access the first month of medication prior to enrollment into payment assistance programs, a model that is not replicable in all settings. DMHC is housed within an integrated safety-net healthcare system that includes primary care as well as infectious diseases and HIV specialty care; this program might be more challenging in a stand-alone STD clinic. On-site pharmacy also is not available at most STD clinics and intensive medication education would be the responsibility of the clinic providers in these settings. Lastly, the follow-up period for this study was 6 months, which is relatively brief and, thus, long-term retention was not captured.

Our study suggests STD clinic-based, same-day PrEP initiation models are highly acceptable, feasible, and safe means of PrEP initiation, and a high proportion of individuals initiated via such models link to ongoing care. STD clinics positioned to initiate but not longitudinally manage individuals on PrEP can utilize patient navigation to support PrEP retention at outside clinics. Rapid PrEP start models are promising approaches to addressing the gap between PrEP need and utilization. More research is needed to assess long-term retention among individuals initiating PrEP under rapid initiation models.

## Supplementary Data

Supplementary materials are available at *Open Forum Infectious Diseases* online. Consisting of data provided by the authors to benefit the reader, the posted materials are not copyedited and are the sole responsibility of the authors, so questions or comments should be addressed to the corresponding author.

ofz310_suppl_supplementary_material
