# Supplementary material for: Same-Day HIV Pre-Exposure Prophylaxis (PrEP) Initiation During Drop-in Sexually Transmitted Diseases Clinic Appointments Is a Highly Acceptable, Feasible, and Safe Model that Engages Individuals at Risk for HIV into PrEP Care
Source: Open Forum Infect Dis. 2019 Jun 27;6(7):ofz310. doi: 10.1093/ofid/ofz310 (PMC6641790; doi:10.1093/ofid/ofz310)
Supplement: ofz310_suppl_supplementary_material [file ofz310_suppl_supplementary_material.docx]

**Supplemental Material**

*Same-day PrEP during STD Clinic visits*

Kamis et al

| **Client Satisfaction Survey** | N (%) |
| --- | --- |
| **1. Did you like having the option of getting PrEP on the same day that you came to the STD clinic?** |  |
| Yes  No | 54 (100%)  0 (0%) |
| **Comments: ______________________________* |  |
| **2. Would you prefer to receive PrEP services directly in the STD clinic rather than in the 5^th^ floor infectious disease clinic?** |  |
| Yes  No | 23 (53%)  20 (47%) |
| **Comments: _______________________________* |  |
| **3. Did you have any problems starting PrEP?** |  |
| Yes  No | 7 (13%)  45 (87%) |
| **Comments: _______________________________* |  |
| **4. In the last 7 days, how many days did you take PrEP?** | Median = 7 days  IQR 6-7 days |
| **5.1 Do you have a primary care provider?** |  |
| Yes  No | 27 (51%)  26 (49%) |
| **5.2 If yes, is this primary care provider new in the last 3 months?** |  |
| Yes  No | 13 (48%)  14 (52%) |
| **6.1 Do you have insurance?** |  |
| Yes  No | 36 (68%)  17 (32%) |
| **6.2 If yes, is this insurance coverage new in the last 3 months?** |  |
| Yes  No | 7 (21%)  27 (79%) |
| **7. *How do you think that we could improve our PrEP services? : _______________________________** | |
| **8. Do you plan to continue PrEP to prevent HIV infection?** |  |
| Yes  No | 49 (96%)  2 (4%) |
| **9. *Additional Comments: _______________________________** |  |
| ******Note:*** *Qualitative responses not reported here.* |  |
